# Endophytic *Beauveria bassiana* in maize: influence of genotype, fungal source, inoculation methods, and time on colonization and fitness of Fall armyworm

**DOI:** 10.1038/s41598-026-44290-1

**Published:** 2026-03-24

**Authors:** Tamegnon Hospice Tossou, Elie Ayitondji Dannon, A. Sylvia S. Schleker, Cyriaque Agboton, Ouorou Kobi Douro-Kpindou, Florian M. W. Grundler, Christian Borgemeister, Manuele Tamò

**Affiliations:** 1 Center for Development Research (ZEF), Ecology and Natural Resource Management, University of Bonn, Genscherallee 3, 53113 Bonn, Germany; 2 International Institute of Tropical Agriculture (IITA), Biorisk Management Facility (BIMAF), IITA-Benin, 08 BP 0932 Tri Postal, Cotonou, Republic of Benin; 3 Institute of Crop Science and Resource Conservation (INRES), Molecular Phytomedicine (MPM), University of Bonn, Karlrobert-Kreiten-Str. 13, 53115 Bonn, Germany

**Keywords:** Maize genotype, *Beauveria bassiana*, *Spodoptera frugiperda*, Endophyte, Ecology, Ecology, Microbiology, Plant sciences

## Abstract

We investigated how *Beauveria bassiana* isolate origin and maize genotype affect endophytic colonization and suppression of the Fall armyworm (FAW). The objectives were to determine whether isolate origin (insect or soil-derived) influences colonization across maize tissues, assess the role of four genotypes (two landraces and two improved lines), and evaluate FAW fitness on colonized plants. Three indigenous isolates, two insect-derived (Bb11, Bb115) and one soil-derived (DL1.1), were applied to four maize genotypes. These included two landraces (Kokoli Daneri, Ovinonboe) and two improved varieties (Faaba QPM, TZL Composite 4W Benin). Inoculation was performed using foliar spraying and seed coating. Colonization declined over time but varied significantly with isolate, genotype, and tissue type (*p* < 0.0001). Insect-derived isolates achieved higher colonization, particularly in stems and roots of landraces, indicating genotype-dependent compatibility. Bb115 achieved systemic colonization in Kokoli Daneri leaves at 7 days after inoculation. Feeding assays showed that FAW larvae fed on colonized plants had reduced survival, lower larval and pupal weights, and reduced adult fecundity and emergence. FAW fitness was assessed using corrected larval mortality, larval and pupal weight and size, adult emergence, and reproduction (adult emergence, fecundity). The reduction of fitness remained moderate and was more pronounced in landrace and insect isolate combinations. These results emphasize the importance of fungal isolate, maize genotype, inoculation method, and timing in enhancing endophytic biocontrol.

## Introduction

*Spodoptera frugiperda* J.E. Smith (Lepidoptera: Noctuidae), commonly known as the Fall armyworm (FAW), is a major pest of maize that causes substantial yield losses in Africa and other maize-growing regions^1,2^. Female moths locate maize plants using plant volatiles and lay eggs primarily on the underside of leaves or occasionally on stems^3–5^. Upon hatching, larvae feed on leaves, producing characteristic damage patterns such as pinholes, window-like patches, and shredded edges^6^. FAW larvae feed on leaves, tassels, and cobs, producing considerable damage and reducing grain yield^7^. Their cryptic feeding behavior within whorls and stems complicates control with surface-applied insecticides^8^.

Maize landraces often exhibit inducible defense mechanisms, shaped by long-term adaptation to local environments and insect pressures^9^. For instance, the authors identified a terpene synthase gene (TPS23) in a maize landrace that is specifically activated by herbivore egg deposition, a response absent in elite inbred lines. Domestication and breeding have shifted maize from dynamic inducible defenses toward more passive, constitutive forms, potentially limiting defense flexibility^10^. Landraces can detect herbivore-associated cues, such as proteins in insect frass, triggering targeted defenses that reduce herbivore fitness^11^. Herbivore-induced plant volatiles (HIPVs), including indole and (E)-DMNT, not only enhance plant defenses but also attract beneficial insects like parasitoids^12^. For example, the maize landrace Nyamula releases volatiles after egg-laying that prime nearby plants, a trait largely absent in modern hybrids, and its HIPVs attract the stemborer parasitoid *Chelonus bifoveolatus* Szepligeti (Hym.: Braconidae) more effectively than hybrid varieties^13^. However, compared to landraces, modern genotypes show weaker inducible responses, likely due to the loss or partial retention of defense genes during breeding for yield and uniformity^14–16^. Recombination and linkage drag can disrupt QTL regions controlling inducible defenses^15,17^. Consequently, landrace cultivars often resist pests naturally and require fewer chemical inputs, while improved varieties yield more but are more vulnerable to herbivores and rely more on synthetic insecticides^18,19^. This trade-off highlights the need for varieties that combine high yield potential with natural pest resistance, creating an opportunity for complementary biocontrol. Endophytic entomopathogenic fungi (EPFs) offer one such solution.

*Beauveria bassiana* endophytes represent such a promising complementary control strategy. Since its first report as an endophyte in food crops^20^, *B. bassiana* has been shown to colonize a wide range of plant species, including cocoa^21^, cotton^22^, oilseed rape^23^, beans^24^, tomato^25^, and maize^26^. However, evidence for negative effects on FAW has been demonstrated primarily in maize systems, where endophytic *B. bassiana* reduces FAW survival and larval performance once established in plant tissues^27^.

Despite these advances, important gaps remain in our understanding of *B. bassiana* as a maize endophyte. Most studies have focused on isolate virulence or inoculation techniques in isolation, with limited attention to integrated host–fungus–insect interactions^20,27^. Emerging evidence suggests that fungal isolate origin may play a critical role in endophytic behavior and biocontrol efficacy: insect-derived isolates often exhibit stronger associations with herbivores than soil-derived strains^23,28^. However, this distinction has been poorly examined in maize systems, particularly in relation to tissue-specific colonization and subsequent effects on FAW fitness. Maize genotypes also differ substantially in defense traits, especially between landraces and improved varieties; however, their compatibility with endophytic EPFs and the consequences for insect suppression remain largely unresolved^14–16^. Furthermore, factors such as inoculation method and time after inoculation influence *B. bassiana* establishment and persistence within plant tissues^25,26^, yet these variables are rarely evaluated simultaneously across multiple genotypes and isolate origins.

Endophytic colonization has been associated with enhanced plant growth and stress tolerance, as well as suppression of herbivorous insects. However, in maize, most studies have emphasized establishment success and larval mortality, with limited assessment of how colonization varies across tissues or influences broader insect fitness traits. Finally, although reduced FAW survival on endophyte-colonized maize has been documented^27^, comprehensive assessments linking endophytic colonization to multiple insect fitness parameters, including larval growth, adult emergence, and reproductive potential, are still limited.

In this context, the present study addresses these knowledge gaps by systematically evaluating the interaction between maize genotype, *B. bassiana* isolate origin, and FAW performance. Specifically, we aimed to: (i) evaluate whether isolate origin (insect or soil) affects *B. bassiana* colonization across maize tissues, (ii) examine how maize genotype (landraces or improved varieties) influences colonization dynamics, and (iii) assess the fitness of FAW fed on endophyte colonized maize**.**

## Results

### Verification of fungal colonization (PCR and culture-based detection on PDA)

Surface sterilization of seeds was effective, as no microbial growth was observed from the final rinse water following incubation, confirming the removal of epiphytic contaminants. Foliar application of 3 mL of a *B. bassiana* conidial suspension (1 × 10⁸ conidia mL^−1^) successfully established endophytic colonization in maize plants. Molecular analysis using PCR confirmed the presence of the inoculated fungal isolates in leaves, stems, and roots at 7 DAI in all treated varieties, whereas no fungal DNA was detected in non-inoculated control plants. These PCR results were fully consistent with culture-based detection on PDA, showing 100% concordance between the two methods at 7 DAI.

**Fig. 1:**
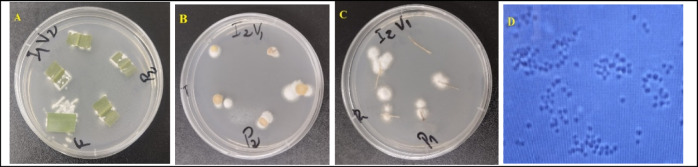
Systemic colonization of Kokoli Daneri inoculated with *Beauveria bassiana* strain Bb115. (**A**) Leaf, (**B**) stem, and (**C**) root tissues at 7 DAI, (**D**) image showing spores of *B. bassiana* strain Bb115.

Culturing plant organs on PDA proved to be a reliable method for detecting fungal endophytes (Fig. 1). Fungal colonization was confirmed in leaves, stems, and roots across all sampling dates, except at 21 DAI in the local variety Ovinonboe inoculated with Bb11, where agar plating did not detect the fungus. Morphological examination of conidia, conidiophores, and mycelia (Fig. 1A–D) further facilitated the identification of *B. bassiana* both from plant tissues and from mycosed larval cadavers. None of the inoculated fungal isolates were recovered from control plants, whereas all treated plants consistently yielded the expected endophytes, demonstrating the reliability of both molecular and culture-based detection methods in confirming successful colonization.

Additionally, several other fungal species were isolated from both control and inoculated plants, indicating the presence of native seed-borne endophytes. These non-target fungi were identified based on morphological characteristics using stereomicroscopy provided with camera. The landrace maize varieties*,* Kokoli Daneri and Ovinonboe, harbored a diverse community of native antagonistic fungi, including *Aspergillus flavus*, *Fusarium oxysporum*, *Verticillium* spp., *Penicillium* spp., *Chaetomium* spp., *Epicoccum* spp., *Phoma* spp., and *Chrysosporium* spp. In contrast, the improved maize varieties, Faaba-QPM and TZL Composite 4W Benin, exhibited a less diverse native fungal community, primarily consisting of *Phoma* spp., *Chaetomium* spp., and *Lasiodiplodia theobromae*.

### Colonization dynamics of *Beauveria bassiana* by tissue and genotype

#### Leaf colonization

**Fig. 2:**
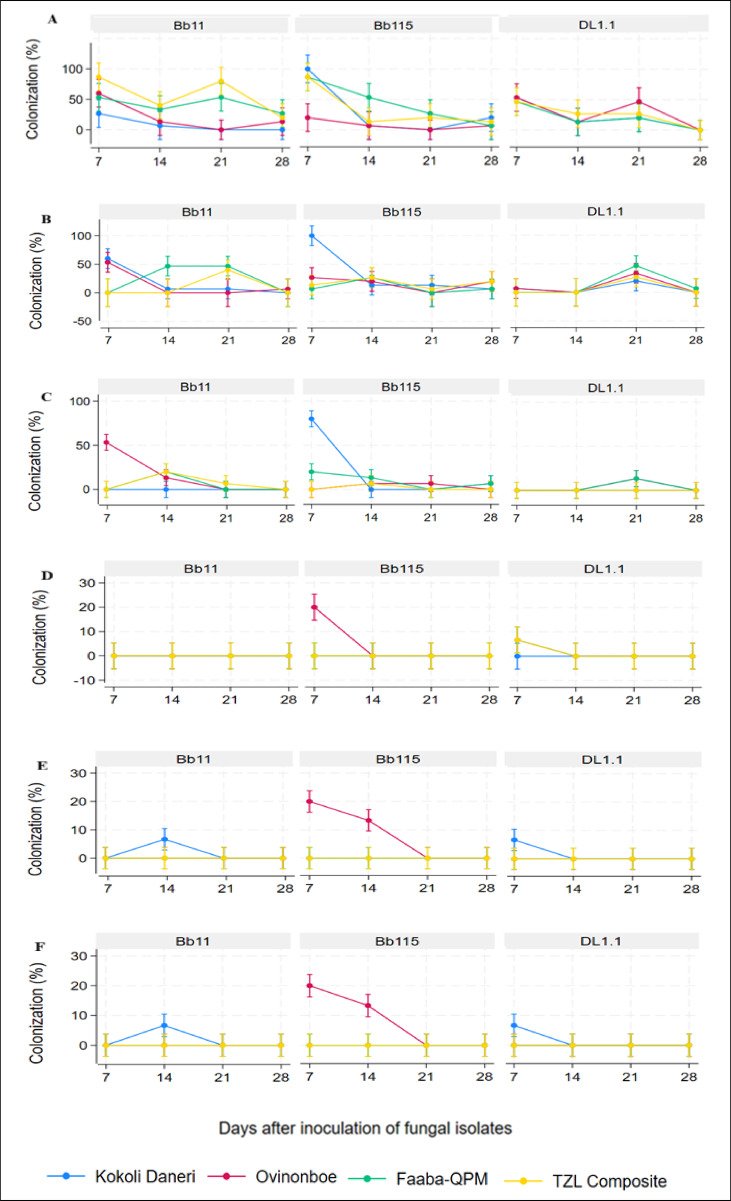
Trends in maize colonization over time. Panels (**A** to **C**) show colonization in leaves, stems, and roots, respectively, following spray inoculation, while panels (**D** to **F**) show colonization in leaves, stems, and roots, respectively, following seed-coating inoculation.

Leaf colonization of *B. bassiana* through foliar spraying varied considerably over time and among maize genotypes (Fig. 2A). The improved varieties, TZL Composite 4 W Benin showed high colonization with Bb11 at 7 DAI (87%), which declined to 40% at 14 DAI, increased again to 80% at 21 DAI, and dropped to zero by 28 DAI. Faaba-QPM followed a similar pattern, with Bb11 reaching 53% at 7 DAI, declining to 33% at 14 DAI, rising to 53% at 21 DAI, and disappearing by 28 DAI. Among local varieties, Ovinonboe exhibited higher colonization than Kokoli Daneri at 7 DAI, while both varieties showed similar levels from 14 to 28 DAI. Bb115 reached 100% colonization in Kokoli Daneri at 7 DAI, decreased sharply to 7% at 14 DAI, dropped to zero at 21 DAI, and partially recovered to 20% at 28 DAI. DL1.1 showed moderate leaf colonization across varieties, with Ovinonboe showing the highest levels at 7 and 21 DAI. Overall, Bb115 consistently outperformed Bb11 and DL1.1 in early stages (7–14 DAI), whereas Bb11 showed transient high colonization in improved varieties and DL1.1 exhibited moderate, variable patterns.

#### Stem colonization

Stem colonization patterns also exhibited tissue- and genotype-specific differences (Fig. 2B). In local varieties, Bb11 initially reached 60% at 7 DAI but declined sharply to 5% by 14 DAI. Bb115 peaked at 100% in Kokoli Daneri at 7 DAI, whereas DL1.1 peaked later at 21 DAI in the same genotype. Colonization in improved varieties was generally lower and more variable across time points for all isolates. These results indicate that Bb115 had the most consistent and rapid establishment in stems, particularly in local genotypes, while other isolates displayed delayed or sporadic colonization.

#### Root colonization

Root colonization showed distinct dynamics compared with leaves and stems (Fig. 2C). The Bb11 was largely absent at 7 DAI in most varieties, except for 50% colonization in Ovinonboe. Bb115 achieved the highest root colonization at 7 DAI in Kokoli Daneri (80%) and Faaba-QPM (25%), whereas DL1.1 displayed moderate colonization across all varieties, with relatively better establishment in roots than in other tissues.

Seed coating primarily enhanced colonization in local varieties, with Bb115 establishing consistently in Kokoli Daneri and Ovinonboe across leaves at 7 DAI and in stems and roots by 14 DAI (Fig. 2D, E, and F). By contrast, colonization in improved varieties was sporadic and less successful, particularly for DL1.1.

### Selection of promising isolate–genotype combinations for bioassays

Across all tissues and genotypes, Bb115 consistently exhibited the highest colonization efficiency, achieving 100% in Kokoli Daneri and 87% in Faaba-QPM at 7 DAI. Bb11 showed transient high colonization in improved varieties, while DL1.1 demonstrated moderate and variable patterns, with better establishment in roots. The combinations Kokoli DanerixBb115 and Faaba-QPMxBb115, especially when applied via leaf spray, emerged as the most promising for subsequent bioassays against FAW.

## Effects on FAW mortality, development, and reproduction.

### Influence of genotype, isolate, inoculation method, and time (Table 1)

**Table 1:** Mixed-effects maximum likelihood regression models for the effects of fungal isolate, inoculation method, maize genotypes, and time post-inoculation on colonization (%) of maize organs (leaves, stems, and roots) by entomopathogenic fungi as endophytes at 7, 14, 21, and 28 DAI.

		Maize leaves			Maize stems			Maize roots		
	df	chi2	*P* > chi2	Coef	chi2	*P* > chi2	Coef	chi2	*P* > chi2	Coef
Isolate	3	0.71	0.7019(ns)		78.98	0.0001***		172.5	0.0001***	
Genotype	3	57.64	0.0001***		33.08	0.0001***		96.42	0.0001***	
Inoculation	1	1228.5	0.0001***		536.5	0.0001***		39.01	0.0001***	
Isolate x Genotype	6	83.69	0.0001***		51.71	0.0001***		126.6	0.0001***	
Isolates x Inoculation	2	7.95	0.0188*		30.04	0.0001***		10.49	0.0053***	
Genotype x Inoculation	3	54.79	0.0001***		25.62	0.0001***		62.27	0.0001***	
Isolate x Genotype x Inoculation	6	114.77	0.0001***		50.21	0.0001***		129.5	0.0001***	
Time	3	412.72	0.0001***	-3.4	102.2	0.0001***	-10.3	143.1	0.0001***	-0.01

*** *p* < 0.0001; *p < 0.05; ns = not significant at α = 0.05.

*Influence of maize genotype on endophytic colonization* maize genotype significantly influenced colonization of *B. bassiana* across all tissues. Mixed-effects regression revealed strong genotype effects on leaves (χ^2^ = 57.64, *p* < 0.0001), stems (χ^2^ = 33.08, *p* < 0.0001), and roots (χ^2^ = 96.42, *p* < 0.0001). These results indicate that host genetic background is a key determinant of successful fungal establishment.

*Effect of fungal isolate on tissue-specific colonization* fungal isolate alone had tissue-specific effects on endophytic colonization. Isolate origin significantly influenced stem (χ^2^ = 78.98, *p* < 0.0001) and root colonization (χ^2^ = 172.5, *p* < 0.0001), but not leaf colonization. This indicates that the origin of the fungal isolate may play an important role, particularly in internal tissues, and that its effectiveness cannot be generalized across all maize organs.

*Impact of inoculation methods* Inoculation methods were the strongest main factor influencing colonization across tissues. Leaves (χ^2^ = 1228.5, *p* < 0.0001), stems (χ^2^ = 536.5, *p* < 0.0001), and roots (χ^2^ = 39.01, *p* < 0.0001) all showed significant differences depending on the inoculation technique used. These findings suggest that method optimization is critical for achieving high and consistent endophytic establishment.

*Interactions between factors* interactions among isolate, genotype, and inoculation methods were significant and tissue-dependent.

Isolate × Genotype interactions were highly significant in leaves (χ^2^ = 83.69), stems (χ^2^ = 51.71), and roots (χ^2^ = 126.6; all *p* < 0.0001).

Genotype × Inoculation interactions also significantly affected colonization in leaves (χ^2^ = 54.79), stems (χ^2^ = 25.62), and roots (χ^2^ = 62.27; all *p* < 0.0001).

Isolate × Inoculation interactions were weaker but still significant (leaves: χ^2^ = 7.95, *p* = 0.0188; stems: χ^2^ = 30.04, *p* < 0.0001; roots: χ^2^ = 10.49, *p* = 0.0053).

The three-way interaction (Isolate × Genotype × Inoculation) was highly significant in leaves (χ^2^ = 114.77), stems (χ^2^ = 50.21), and roots (χ^2^ = 129.5; all *p* < 0.0001), highlighting the complex interplay between host genetics, fungal origin, and application methods in determining colonization success.

### Effects of time post-inoculation

Time post-inoculation significantly influenced colonization in all tissues. Leaves (χ^2^ = 412.72, *p* < 0.0001) and stems (χ^2^ = 102.2, *p* < 0.0001) showed a gradual decline in colonization over time, whereas root colonization remained relatively stable (χ^2^ = 143.1, *p* < 0.0001; regression coefficients: leaves − 3.4, stems − 10.3, roots − 0.01). This indicates that persistence of *B. bassiana* varies by tissue type, which has implications for timing in biocontrol applications.

### Corrected larval mortality and impaired development of Fall armyworm on fungal-colonized and non-colonized maize

#### Corrected mortality trend of Fall armyworm larvae

**Fig. 3:**
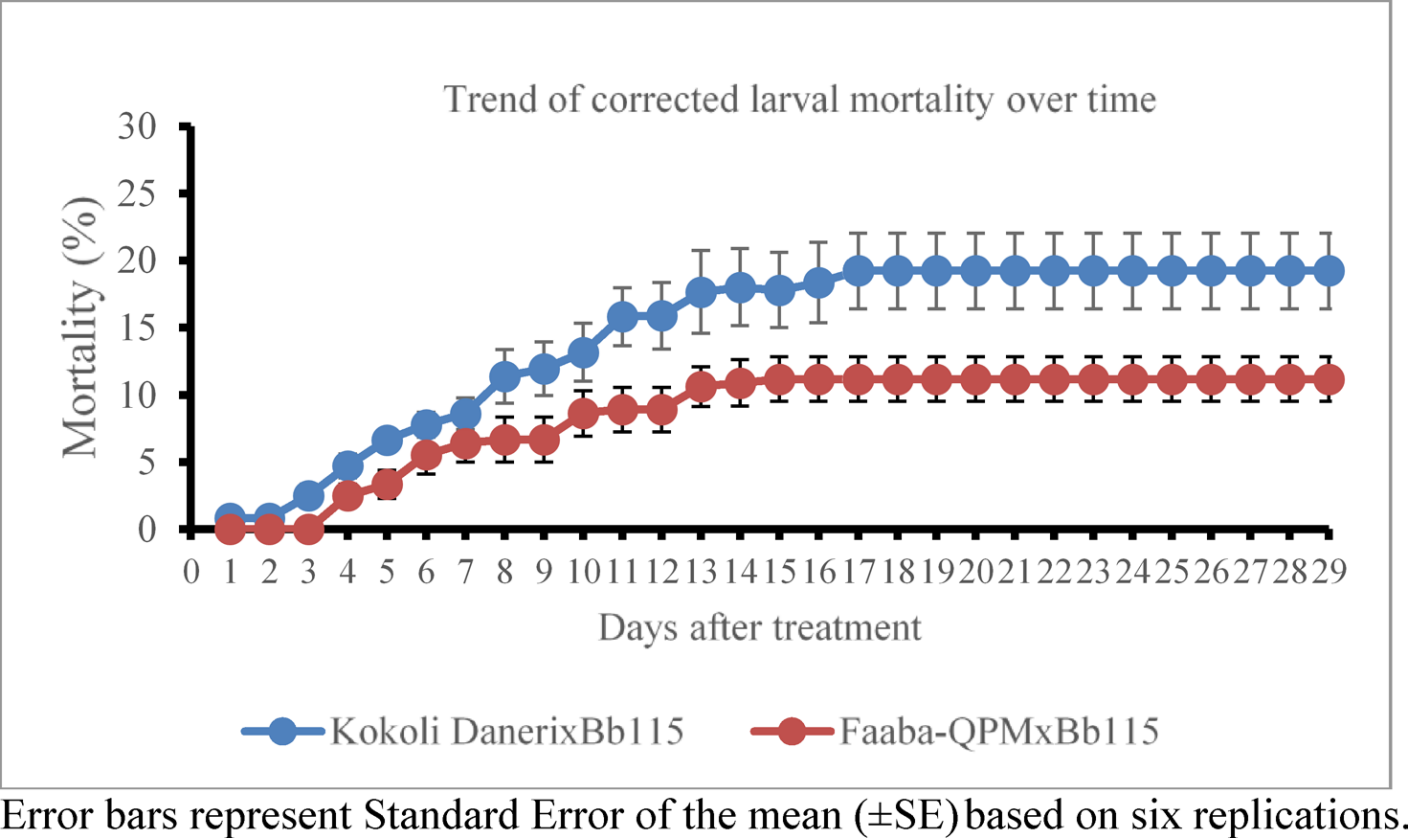
Trends in Fall armyworm corrected larval mortality over time as a result of a *Beauveria bassiana* strain Bb115 inoculation.

**Fig. 4:**
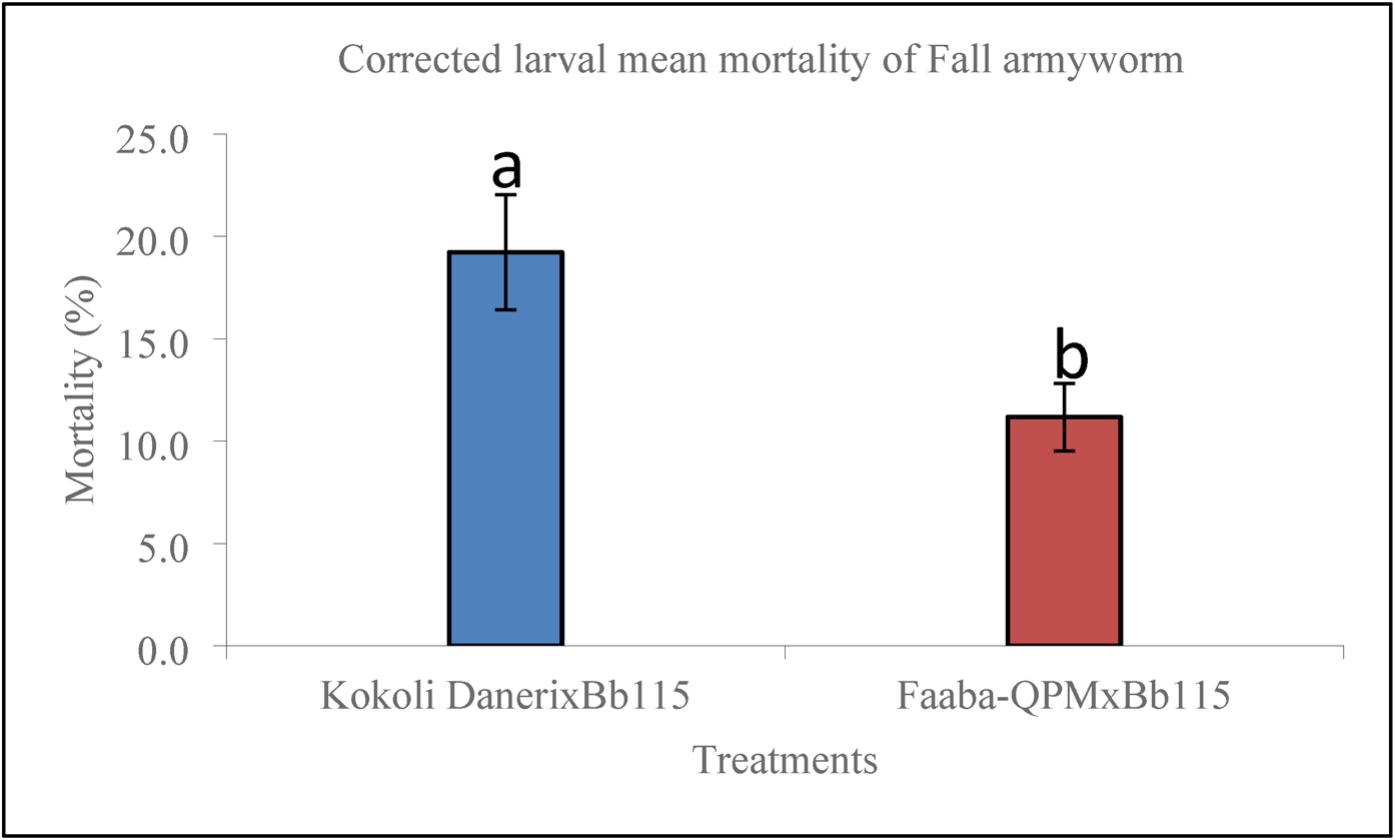
Corrected larval mean mortality of Fall armyworm following treatment with *Beauveria bassiana* strain Bb115. Kokoli DanerixBb115 = Inoculated Kokoli Daneri; Faaba-QPMxBb115 = Inoculated Faaba-QPM.

Monitoring of larval mortality revealed a clear and sustained effect of *B. bassiana* colonization (Fig. 3). In Kokoli Daneri inoculated with Bb115, FAW mortality began as early as 1 to 3 DAI and increased steadily up to about 17 DAI, after which it plateaued at approximately 19.2%. In Faaba-QPM treated with the same isolate, mortality started later (around 4 DAI), rose gradually until 13 to 15 DAI, and then stabilized at about 11.2%. The corrected mortality levels were consistently higher in Kokoli Daneri than in Faaba-QPM throughout the observation period, indicating superior effects of Bb115 in Kokoli Daneri. The corrected mean mortality of FAW larvae differed significantly between maize varieties inoculated with *B. bassiana* Bb115 (Fig. 4). Larvae fed on Kokoli Daneri × Bb115 exhibited a moderately higher corrected mean mortality (19.2%) than those fed on Faaba-QPM × Bb115 (11.2%).

### Effects of fungal treatment on the development of Fall armyworm

Fungal inoculation of maize with *B. bassiana* had significant effects on the development, survival, and reproduction of FAW across larval, pupal, and adult stages (Tables 2 and 3). Overall treatment effects were highly significant (*p* < 0.01), indicating that endophytic colonization influenced multiple fitness-related traits throughout the insect life cycle. These effects were consistently observed across both maize genotypes, although their magnitude varied between Kokoli Daneri and Faaba-QPM.

**Table 2:** Mean weight and length (± SE) of Fall armyworm developmental stages on Kokoli Daneri and Faaba-QPM maize under laboratory conditions following treatment with *Beauveria bassiana* strain Bb115.

Developmental stage	Kokoli DanerixWater	Kokoli DanerixBb115	Faaba-QPMxWater	Faaba-QPMxBb115
L2—Weight (mg)	29.2 ± 1.0 ᵃ	27.7 ± 1.2 ᵃ	36.6 ± 1.2 ᵇ	36.8 ± 1.2 ᵇ
L3—Weight (mg)	97.5 ± 1.7 ᶜ	73.8 ± 2.9 ᵃ	108.7 ± 1.7 ᵈ	84.5 ± 2.7 ᵇ
L4—Weight (mg)	137.5 ± 3.9 ᵇ	105.9 ± 3.7 ᵃ	131.4 ± 1.7 ᵇ	107.6 ± 3.5 ᵃ
L5—Weight (mg)	201.4 ± 29.3 ᶜ	155.0 ± 69.5 ᵃ	197.9 ± 5.8 ᶜ	170.2 ± 5.1 ᵇ
L6—Weight (mg)	216.0 ± 3.3 ᵈ	156.1 ± 6.4 ᵃ	200.0 ± 3.2 ᶜ	162.7 ± 5.4 ᵃ
Pupa—Weight (mg)	98.2 ± 1.0 ᶜ	75.9 ± 2.7 ᵇ	96.0 ± 0.7 ᶜ	65.1 ± 2.1 ᵃ
Pupa—Length (mm)	12.0 ± 0.1 ᵇ	10.1 ± 0.4 ᵃ	12.4 ± 0.4 ᵇ	9.5 ± 0.4 ᵃ

L = larval instar. Means within each row followed by the same letter are not significantly different (ANOVA followed by Tukey’s HSD, *p* < 0.05). “xBb115” indicates maize inoculated with the fungus.

**Table 3:** Developmental performance and survival of Fall armyworm larvae fed on *Beauveria bassiana*–colonized and non-inoculated maize leaves under laboratory conditions.

Developmental Stage of FAW	Kokoli DanerixWater	Kokoli DanerixBb115	Faaba-QPMxWater	Faaba-QPMxBb115
Mean initial larvae (%)	100% (20/20)	100% (20/20)	100% (20/20)	100% (20/20)
Mean dead larvae (%)	3.1% (1/20) ᵃ	21.7% (4/20) ᵈ	0.0% (0/20) ᵇ	11.7% (2/20) ᶜ
Mean survived larvae (%)	96.9% (19/20) ᶜ	78.3% (16/20) ᵃ	100% (20/20) ᵈ	88.3% (18/20) ᵇ
Mean pupa (%)	95.8% (19/20) ᵇ	73.8% (15/20) ᵃ	97.5% (19.5/20) ᵇ	72.1% (14.5/20) ᵃ
Mean adult males emerged	16.5 ± 0.6 ᵇᶜ	14.0 ± 0.9 ᵃᵇ	18.3 ± 1.2 ᶜ	12.2 ± 0.9 ᵃ
Mean adult females emerged	21.8 ± 0.7 ᵇ	14.3 ± 1.3 ᵃ	20.7 ± 1.1 ᵇ	13.3 ± 1.4 ᵃ
Mean eggs per female	30.0 ± 2.6 ᵇ	18.0 ± 3.5 ᵃ	32.6 ± 2.7 ᵇ	17.4 ± 2.5 ᵃ

Means within each row followed by the same letter are not significantly different (ANOVA followed by Tukey’s HSD, *p* < 0.05). “xBb115” indicates maize inoculated with the fungus. Kokoli DanerixWater and Faaba-QPMxWater are uninoculated plants.

### Larval growth and development

Larval growth was significantly suppressed in FAW fed on *B. bassiana*-inoculated maize compared to controls across most instars (L2–L6) (Table 3). The strongest effects were observed at intermediate and late instars, as reflected by high F-values for larval weight at L3 (F = 73.44, *p* < 0.001) and L6 (F = 33.31, *p* < 0.001). Across all instars, larvae feeding on inoculated plants exhibited lower mean weights than those feeding on non-inoculated plants. For example, at the L6 stage, larval weight on inoculated Kokoli Daneri plants declined from 216.0 ± 3.3 in controls to 156.1 ± 6.4 mg, while a similar reduction was observed on Faaba-QPM plants, from 200.0 ± 3.2 to 162.7 ± 5.4 mg.

### Pupal traits

Negative effects of fungal treatment persisted into the pupal stage. Both pupal weight and pupal length were significantly reduced in FAW reared on inoculated maize (Table 3), with pupal weight showing one of the strongest treatment responses (F = 83.25, *p* < 0.001). On Faaba-QPM, pupal weight declined from 96.0 ± 0.7 mg in the control to 65.1 ± 2.1 mg following inoculation, while pupal length decreased from 12.4 ± 0.4 to 9.5 ± 0.4 mm. Comparable reductions were observed in Kokoli Daneri.

### Survival and adult emergence

Survival from larva to adult was significantly reduced in fungal treatments (Table 4). Corrected larval mortality increased on inoculated plants, while the proportion of larvae reaching the pupal and adult stages declined. For instance, corrected larval mortality increased in the Kokoli DanerixBb115 to 19.2%, and to 11.2% in Faaba-QPMxBb115. Correspondingly, adult emergence was significantly reduced for both sexes, with treatment effects evident for males (F = 8.89, *p* = 0.001) and females (F = 13.64, *p* < 0.001). Adult female emergence on inoculated plants declined to 14.3 ± 1.3 individuals in Kokoli Daneri and 13.3 ± 1.4 in Faaba-QPM, compared to 21.8 ± 0.7 and 20.7 ± 1.1 in the respective controls.

**Table 4:** Effects of fungal inoculation on Fall armyworm oviposition and larval survival on two maize varieties following treatment with *Beauveria bassiana* strain Bb115.

Treatments	Mean egg batches	Survival time
Kokoli DanerixWater	30.0 ± 2.6^b^	11.0 ± 4.1^ab^
Kokoli DanerixBb115	18.0 ± 3.5^a^	9.6 ± 3.4^ab^
Faaba-QPMxWater	32.6 ± 2.7^b^	13.8 ± 3.3^a^
Faaba-QPMxBb115	17.4 ± 2.5^a^	5.0 ± 1.7^b^

### Reproductive output

Reproductive performance was also significantly impaired by fungal treatment (Table 4). Females emerging from larvae fed on inoculated maize laid significantly fewer egg batches than those from control treatments. Egg production per female declined from 30.0 ± 2.6 to 18.0 ± 3.5 on Kokoli Daneri and from 32.6 ± 2.7 to 17.4 ± 2.5 on Faaba-QPM. Adult emergence was also reduced in fungal treatments, with fewer males and females emerging compared with the controls.

### Effects of treatments on Fall armyworm survival and oviposition

Inoculation of maize with *B. bassiana* strain Bb115 significantly affected key survival and reproductive parameters of FAW (Table 4). Oviposition was consistently reduced on inoculated plants across both maize varieties. Females laid significantly fewer egg batches on Kokoli DanerixBb115 (18.0 ± 3.5) and Faaba-QPMxBb115 (17.4 ± 2.5) compared with their respective controls, which recorded 30.0 ± 2.6 and 32.6 ± 2.7 egg batches.

Larval survival was also significantly influenced by fungal treatment. Mean survival time was shortest on Faaba-QPMxBb115 plants (5.0 ± 1.7 days), which was significantly lower than that observed on non-inoculated Faaba-QPM (13.8 ± 3.3 days). In Kokoli DanerixBb115, larval survival was moderately reduced following inoculation (9.6 ± 3.4 days) compared to the control (11.0 ± 4.1 days), although these differences were less pronounced.

Please, the following footnote should be under Table 4. "Values represent the mean number of egg batches per plant and the mean larval survival time (days) of FAW on inoculated and uninoculated maize varieties. Within each column, means sharing the same letter are not significantly different (Tukey’s HSD, *p* < 0.05). Kokoli DanerixWater and Faaba-QPMxWater are uninoculated plants."

## Discussion

### Main findings: endophytic *Beauveria bassiana* as a biocontrol agent against Fall armyworm

This study demonstrates that endophytic colonization of maize by *B. bassiana*, particularly strain Bb115, can significantly impair the fitness of FAW by reducing oviposition, larval survival, and overall development. These findings corroborate previous reports that EPFs, when established as endophytes, suppress herbivorous insects primarily through plant-mediated effects rather than direct infection^26,27,29^. Importantly, the present results link successful endophytic colonization with measurable negative impacts on FAW reproduction and survival, reinforcing the role of endophytic EPFs as complementary tools in FAW management.

### Influence of inoculation methods on endophytic establishment

Both foliar application and seed coating successfully established systemic endophytic colonization of maize, confirming that multiple delivery pathways can be effective. Foliar application resulted in rapid and widespread colonization of aerial tissues, particularly leaves, consistent with previous studies demonstrating efficient penetration through stomata or cuticular micro-openings^25,26^. In contrast, seed coating favored colonization of belowground tissues and provided more consistent establishment in local maize varieties, suggesting that early root-associated colonization may enhance systemic spread. These results indicate that inoculation methods strongly shape the spatial and temporal distribution of endophytic *B. bassiana*, a factor that should be considered when optimizing field applications.

### Genotype-dependent effects on Fall armyworm fitness

The level of FAW suppression varied between maize genotypes, highlighting the importance of host plant traits in determining endophyte efficacy. In Faaba-QPM, Bb115 inoculation significantly reduced larval survival time, whereas in the landrace Kokoli Daneri the reduction was weaker and not statistically significant. Such genotype-dependent responses have been reported in other plant–endophyte–insect systems and may reflect differences in defense chemistry, tissue structure, or nutrient composition^30–33^. These findings suggest that maize breeding history influences not only pest susceptibility but also compatibility with beneficial endophytes.

### Oviposition deterrence and plant-mediated mechanisms

A high reduction in FAW oviposition was observed on *B. bassiana*-colonized plants, indicating that endophytic infection altered plant cues used by females for host selection. Because adults did not contact the fungus directly, these effects are likely mediated by changes in plant chemistry, such as modified volatile organic compound (VOC) profiles or surface metabolites^34^. Similar endophyte-induced oviposition deterrence has been reported in cotton, where fungal colonization altered VOC emissions and reduced egg laying by *Helicoverpa zea*^35^. This indirect suppression of reproduction suggests that endophytic *B. bassiana* can reduce FAW population growth even before larval feeding begins.

### Temporal dynamics and decline in detectable colonization

Although PCR and culture-based methods confirmed early establishment of *B. bassiana*, culture-based recovery declined over time in several genotype–isolate combinations. This decline does not necessarily indicate fungal elimination but rather reflects biological and methodological constraints. Culture-based detection depends on viable fungal propagules in sampled tissues and may underestimate colonization when fungal biomass becomes sparse, patchy, or metabolically inactive^36,37^. Similar discrepancies between molecular detection and culturing have been reported in other endophyte studies^38,39^.

Several biological mechanisms may explain this temporal decline. Following initial colonization, plant immune responses may restrict fungal proliferation through systemic signaling and the accumulation of antimicrobial metabolites, resulting in low-density or quiescent fungal populations^40,41^. In addition, tissue maturation processes such as lignification and suberization can create physical and chemical barriers that limit fungal growth in older tissues^30,31^. Thus, declining recovery rates likely reflect shifts in fungal distribution and activity rather than complete loss of endophytic presence.

### Effects of fungal isolate origin on colonization persistence

Fungal ecological origin strongly influenced colonization dynamics. Insect-derived isolates (Bb11 and Bb115) exhibited rapid early colonization, particularly in stems and roots, but declined sharply after 14 to 21 days, especially in landrace varieties. In contrast, the soil-derived isolate DL1.1 established more slowly but persisted longer, particularly in improved genotypes. These contrasting patterns suggest a trade-off between early colonization intensity and long-term persistence, as previously reported for EPFs with different ecological backgrounds^42,43^. Such isolate-specific behaviors highlight the importance of selecting fungal strains not only for virulence but also for compatibility with host tissues and persistence over time.

### Tissue-specific colonization and host–fungus interactions

Colonization dynamics varied highly among maize tissues. Leaf colonization peaked early and declined rapidly, whereas stems and roots supported longer persistence, particularly in improved genotypes. Regression analyses indicated stronger declines in leaves and stems than in roots, suggesting that roots may serve as more stable reservoirs for endophytic fungi. Significant three-way interactions among fungal isolate, maize genotype, and inoculation methods underscore the complexity of endophyte establishment and indicate that colonization outcomes are determined by multiple interacting factors rather than single drivers^43^.

### Role of native endophytic communities

Differences in native endophytic communities likely contributed to genotype-dependent persistence of *B. bassiana66*. Landrace varieties harbored a diverse assemblage of fungi, including antagonistic taxa such as *Aspergillus*, *Fusarium*, *Penicillium*, *Chaetomium*, *Epicoccum*, *Phoma*, and *Chrysosporium* spp., several of which produce metabolites known to inhibit EPF growth67^42,44–47^. In contrast, improved varieties exhibited lower endophyte diversity, potentially reducing competitive pressure and allowing greater persistence of introduced fungi. These findings emphasize that resident microbiomes can strongly influence the success of endophytic biocontrol agents.

### Limitations and future directions

This study was conducted under controlled greenhouse conditions, which only partially reflect field environments where abiotic stress, microbial competition, and fluctuating climate could affect *Beauveria bassiana* colonization and FAW suppression^27^. While we used a combination of culture-based detection and species-specific PCR to confirm colonization, molecular verification was performed only at 7 DAI. Although culture-based re-isolation continued for up to 28 DAI, this approach may underestimate long-term persistence and the confirmation of systemic colonization, particularly in tissues where endophytes exist at low abundance or in a quiescent state. Future studies may use sequencing, quantitative PCR, fluorescence microscopy, or fungal biomass markers to provide more sensitive and temporal confirmation of fungal presence. We only used two maize genotypes and one fungal strain (Bb115) in FAW bioassays, limiting generalization across varieties and isolates. In addition, native endophytic communities, which differed between landraces and improved varieties, could influence fungal persistence and biocontrol efficacy in the open field. Mechanisms underlying FAW suppression, whether direct fungal effects or plant-mediated changes, were not measured. Future studies should evaluate field performance, extend molecular monitoring, and examine the biochemical and ecological basis of endophyte-mediated pest suppression.

### Implications for FAW management

FAW continues to cause significant yield losses in African maize. Chemical control may be limited due to insecticide resistance and high costs^48,49^. The ability of endophytic *B. bassiana* to reduce oviposition, impair larval survival, and interact with host plant defenses underscores its potential in integrated pest management. Using compatible maize genotypes, effective fungal strains, and optimized inoculation methods could improve the reliability and sustainability of FAW management within integrated pest management programs.

## Conclusion

This study showed the potential of indigenous *B. bassiana* strains as effective endophytic biocontrol agents against FAW in maize. Colonization success and pest suppression were influenced by fungal origin, maize genotype, and time post-inoculation, with insect-derived strains (Bb11, Bb115) outperforming the soil-derived isolate (DL1.1). The first three weeks after inoculation were critical for establishment and FAW suppression, with local maize varieties, particularly Kokoli Daneri, supporting more effective colonization and moderate reductions in larval growth, survival, and fecundity. These results highlight the importance of matching endophytes with genetically compatible hosts to enhance biocontrol efficacy. Future work should evaluate field performance and explore the biochemical and genetic mechanisms underlying maize responses to endophytic colonization.

## Materials and methods

Two experiments were conducted to assess the colonization potential of three indigenous *B. bassiana* strains in different maize varieties and their effects on the fitness of FAW. In the first experiment, *B. bassiana* was inoculated into maize plants through foliar spray and seed coating. Fungal colonization was confirmed by culturing plant tissues on agar media, followed by Polymerase Chain Reaction (PCR) analysis. The second experiment evaluated the impact of fungal colonization and maize variety on the survival and reproduction of the FAW, comparing insects fed on *B. bassiana*-colonized plants with those fed on non-inoculated controls.

### Study organisms

Maize seeds were obtained from the Benin National Institute for Agricultural Research (INRAB), the regional maize reference center under the West and Central African Council for Agricultural Research and Development^50^. A total of twenty varieties (ten landraces with older genetic backgrounds and ten recently improved lines) were initially screened in order to select four of them for further studies. The selection was based on key agronomic and defense traits, such as stem robustness, susceptibility to FAW damage, presence of anthocyanin at the stem collar, plant height, cob size, and incidence of malformed cobs (Table 5). Selection criteria were guided in part by previous studies^51^.

**Table 5:** Overview of maize varieties with status and growth cycle.

Accession code	Variety name	Status	Growth cycle
GBB-Ma-N-2013–0054	Kokoli Daneri	Landrace	Long cycle
GBB-Ma-S-2013–0153	Ovinonboe	Landrace	Early cycle
GBB-Ma-I-2023–0001	Faaba-QPM	Improved variety	Intermediate cycle
GBB-Ma-I-2023–0002	TZL Composite 4W Benin	Improved variety	Intermediate cycle

Three *B. bassiana* strains were obtained from the International Institute of Tropical Agriculture (IITA), Benin, for this study. These included *B. bassiana* strains Bb11, Bb115, and DL1.1. Strain Bb11 was isolated from *Sesamia calamistis* Hampson (Lep.: Noctuidae) in Benin, Bb115 from *Locusta* spp. (Orth.: Acrididae) in Madagascar, and DL1.1 from soil in Dalsalami Lesso, Burkina Faso, using the *Galleria mellonella* (L.) (Lep.: Pyralidae) baiting method^52^. Pure cultures were established on Potato Dextrose Agar (PDA) supplemented with streptomycin (250 mg/L) and incubated for three weeks under controlled conditions (26 ± 2 °C, 70 to 75% relative humidity, 14:10 h light/dark photoperiod). These isolates were selected because of their distinct ecological origins (insect-associated vs. soil-derived) and have previously demonstrated pathogenicity against FAW in preliminary laboratory experiments (Tossou, unpubl. results), making them promising candidates for evaluating both colonization potential and effects on FAW performance.

### Preparation of fungal suspension

Conidia were harvested from three-week-old cultures of *B. bassiana* isolates grown on potato dextrose agar (PDA)^53^. The conidia were carefully scraped from the culture surface and suspended in 10 mL of sterile distilled water containing 0.01% Tween 80. The suspension was then filtered through sterile muslin cloth into clean test tubes to remove agar fragments. Conidial concentration was adjusted to 1 × 10⁸ conidia/mL using a hemocytometer^54^. Prior to plant inoculation, conidial viability was assessed through a germination test^55^, and only suspensions with a germination rate ≥ 95% were used in the experiments.

### Maize seedling production and inoculation

Seeds from four maize varieties were surface-sterilized by immersing in 2% sodium hypochlorite for 2 min, followed by 70% ethanol for 3 min, and rinsed three times with sterile distilled water. Sterilization success was confirmed by plating a sample from the final rinse onto PDA and observing for microbial growth^56^. Soil used for planting was sterilized at 121 °C for 24 h (1 kg per bag). Each 12 kg-capacity plastic pot was filled with 10 kg of this sterile soil, and drainage holes were provided at the bottom. Three seeds were sown per pot and watered regularly; seedlings were thinned to a single plant per pot two weeks after germination. Pots were arranged in a completely randomized design in a greenhouse.

Seedlings were maintained under controlled conditions (25 °C, 75% relative humidity, 12:12 h light/dark cycle) and fertilized at three weeks of age with approximately 1 g NPK and 0.35 g urea per pot^57^. For foliar inoculation, *B. bassiana* conidial suspensions (1 × 10⁸ conidia/mL) were applied to leaves using a 50 mL handheld sprayer, delivering 3 mL per plant according to the assigned fungal isolate and maize variety^4^. Control plants were sprayed with the same volume of sterile water containing 0.01% Tween 80 to account for any surfactant effects^58^. To prevent contamination of soil and lower stems, aluminum foil was wrapped around the base of each plant and the pot top before spraying. Plants were enclosed in plastic bags for 24 h to maintain humidity and promote fungal establishment, then returned to greenhouse conditions and monitored for four weeks. For the seed immersion treatment, surface-sterilized seeds were soaked in 10 mL of conidial suspension for 24 h, then dried on sterile paper towels inside a laminar flow cabinet for 30 min before planting in 12 kg pots with sterilized soil. Control seeds were treated with the same manner using sterile 0.01% Tween 80. Seedlings were thinned to one plant per pot ^54^.

The experiment was carried using a completely randomized blocks design in a factorial arrangement. Variables included in the model consisted of fungal isolates (three fungal isolates) with water as control, inoculation method (foliar spray vs. seed immersion), and maize genotype (four varieties: two landraces and two improved). In total, 32 treatment combinations were generated. Each combination included 48 individual plants. To monitor colonization over time, 12 plants per treatment were destructively sampled at 7, 14, 21, and 28 DAI^54^.

### Assessment of endophytic colonization in maize by *B. bassiana*

To confirm the successful colonization of maize tissues by different *B. bassiana* strains, two complementary approaches were employed: culturing on PDA and PCR-based molecular detection. Leaf, stem, and root samples were collected from greenhouse-grown plants at 7, 14, 21, and 28 DAI.

Plant tissues were surface-sterilized by immersion in 70% ethanol for 2 min, followed by 55 g/L sodium hypochlorite for 2 min, and rinsed twice with sterile distilled water. Sterilization effectiveness was verified by plating a sample of the final rinse onto PDA and incubating at 25 °C for one week. Sterilized tissue fragments (1 cm^2^ for leaves and 1 cm segments for stems and roots) were dried on sterile paper towels under a laminar flow hood before plating. Fragments from each organ per plant were placed on PDA supplemented with 0.1% antibiotics (0.02 g each of streptomycin and penicillin) to limit bacterial growth. Plates were incubated at 26 ± 2 °C, 70 to 75% relative humidity, and a 12:12 h light/dark cycle for three weeks. *Beauveria bassiana* growth was identified based on colony morphology and microscopic characteristics of conidia. Endophytic colonization frequency was calculated as the proportion of tissue fragments exhibiting fungal growth^54^:

$$\text{Colonization frequency }(\mathrm{\%}) =\frac{\text{Number of colonized fragments}}{\text{Total fragments}}x 100$$

For each sampled plant, the roots, stems, and leaves were sectioned into small fragments. From these, five roots, five stems, and five leaf fragments were randomly selected, sanitized, and placed on PDA medium. A plant was classified as colonized when fungal growth was observed in at least one of the five fragments from a given tissue type.

For molecular confirmation, the remaining tissues were preserved in 80% ethanol, and genomic DNA was extracted using the NucleoBond Plant II HMW DNA kit (Macherey–Nagel, Germany). Species-specific oligonucleotide primers for *B. bassiana* were used for PCR at 7 DAI: Forward 5′-CGGCGGACTCGCCCCAGCCCG-3′ and Reverse 3′-CCGCGTCGGGGTTCCGGTGCG-5′^22^. Given the large number of experimental plants and fragments used in this study, it was not feasible to extract and analyze genomic DNA from all material at every sampling date. Consequently, molecular verification by PCR was performed only at 7 DAI to confirm the successful establishment of the inoculated endophytes using both positive and negative controls. At all sampling dates and across all experimental plants, the presence of *B. bassiana* was assessed using the agar planting method. Maize varieties and fungal strains exhibiting leaf colonization greater than 80% at 7 DAI were selected for subsequent bioassays^59,60^. Concordance between culturing and PCR detection was evaluated^61^: 

$$Concordance rate \left( \% \right) = \frac{Number of concordant results }{{Total number of samples}}x 100$$

where: Concordant results = Number of samples where both PCR and culture were either positive or both negative; Total number of samples = All samples tested (including matching and non-matching results). Here, concordant results were defined as samples that were either positive or negative by both methods. Samples positive by PCR but negative by culturing were still considered colonized, reflecting the higher sensitivity of PCR for detecting low or latent fungal colonization^60^. PCR products were not sequenced. The species-specific primers used here to detect *B. bassiana* had been validated in earlier studies^22^. To ensure reliability, positive controls consisting of genomic DNA from pure *B. bassiana* cultures and negative controls including water blanks and DNA from non-inoculated maize were included. Additionally, primer specificity was confirmed using reference strains from the genus *Beauveria* (*B. bassiana* DSMZ 875, *B. felina* DSMZ 4678, *B. brongniartii* DSMZ 6651) under the same PCR conditions. Endophytic colonization was considered confirmed when PCR results were concordant with fungal re-isolation on PDA. This combination of previously validated primers, experimental controls, and culture-based confirmation allowed us to reliably confirm endophytic colonization without sequencing.

### Effects of endophytic *Beauveria bassiana* colonization in maize on the development and reproduction of FAW

To evaluate the influence of endophytic *B. bassiana* on FAW, one fungal strain was inoculated into two maize varieties (one landrace and one improved) using foliar spraying under greenhouse conditions. Freshly laid FAW eggs were obtained from the IITA-Benin insectary to ensure synchronized hatching and uniform larval development. Eggs were incubated in plastic containers for three to five days at 25 ± 2 °C and 70 to 75% relative humidity under a 12:12 h light/dark photoperiod until hatching^62^. Neonates were starved for 24 h to standardize feeding responses, and only active, healthy larvae were selected. Each larva was placed in an aerated plastic container provided with moist filter paper to minimize desiccation and prevent larval cannibalism.

Two feeding groups were established for each maize variety: one fed with endophyte-colonized leaves (treated) and the other with non-inoculated leaves (control). Each group consisted of six vessels, each holding 20 larvae. The experiment was repeated twice, resulting in a total of 960 larvae across all treatments (4 treatments × 6 replicates × 20 larvae × 2 trials). To verify *B. bassiana* colonization, leaf fragments were cultured on PDA and incubated for one week. One week after inoculation, only leaves showing fungal growth were harvested for larval feeding. Leaves were surface-sterilized and dried on sterile paper towels in a laminar flow hood prior to use. Larvae were fed exclusively on these fresh leaves until reaching maturity. The control group received non-inoculated maize leaves. Early instar larvae were fed 250 to 300 mg of fresh leaves daily, increasing to 500 mg per day from the second instar to accommodate growth. Leaf material was replenished daily, and containers were cleaned to maintain hygiene. Larval development and mortality were monitored daily, with molting confirmed by the presence of shed head capsules. Dead larvae were collected daily, surface-sterilized, and incubated on moist filter paper at room temperature for three weeks to confirm fungal infection. Fungal re-isolation was performed by sampling mycelia and conidia from both internal tissues and larval surfaces, followed by microscopic examination for *B. bassiana* morphological characteristics^63^. Upon pupation, pupae were sexed^64^ and paired (1 male:1 female) in 500 cm^3^ containers for adult emergence. Mated adults were transferred to oviposition cages containing folded paper as an egg-laying substrate and a cotton pad soaked in 10% sugar solution (honey) as a food source. Eggs were collected daily, and adult food was replenished until the female’s death. Mortality was recorded throughout the experiment. For each treatment, a total of 30 mating pairs (derived from surviving pupae across six replicates) were established per trial, resulting in 90 mating pairs per treatment across the three independent trials. Only successfully emerged and normally developed adults were used for fertility assessments. The following parameters were recorded to assess the developmental and reproductive performance of FAW: total number of larvae, larval mortality, number of pupae, adult emergence by sex, number of egg batches per female, larval weight (2nd to 6th instars), pupal weight, and pupal length. These measurements provided indicators of growth, survival, and reproductive fitness under endophyte-colonized versus control conditions.

Larval and pupal weights were recorded as fresh weights using an analytical balance (Mettler Toledo, ± 0.1 mg precision). Larvae were weighed individually at the midpoint of each instar (2nd to 6th instars), approximately 12 h after molting and prior to daily feeding to minimize variation due to gut content. Pupae were weighed within 24 h of pupation. Each weight measurement corresponds to a single individual, and means were calculated per replicate.

### Statistical analysis of data

Colonization data were tested for normality and homogeneity of variances. Percentage data were arcsine square-root transformed prior to the Analysis of Variance. Mixed-effects maximum likelihood regression models included fungus treatment, maize genotype, DAI, and application method as fixed effects, and plant as a random effect. Data were reshaped to long format for repeated measures (Days 7, 14, 21, 28). Models were estimated using expectation–maximization and gradient-based optimization, with significance assessed by Wald chi-square tests. Post hoc contrasts of marginal means evaluated main and interaction effects. When significant three-way interactions were detected, main effects were not interpreted independently. Instead, analyses were stratified by tissue type, and simple-effects analyses were conducted with appropriate adjustment for multiple comparisons. Interaction plots were used to facilitate interpretations of tissue-specific colonization patterns.

The FAW bioassays related to mortality, survival, developmental traits, and reproduction were analyzed after checking normality and variance assumptions. Larval mortality in treated groups was corrected for natural mortality observed in the controls using Abbott’s formula: *Corrected mortality (%)* = *[(Mt − Mc)/(100 − Mc)]* × *100*, where Mt is mortality in the treatment, and Mc is mortality in the control. FAW developmental parameters assessed included corrected larval mortality and survival at each developmental stage (larval and pupal weights), pupal length, adult emergence (male and female), and reproductive output (number of egg batches per female). The values presented in Table 3 represent the means ± standard error, calculated from six replicates per treatment, with 20 larvae per replicate, over two experiments. Larval mortality, survival, pupation, and adult emergence of males and females were determined for each treatment. Corrected mortality means were compared using ANOVA at the 5% significance level, while means for the remaining developmental parameters were separated using Tukey’s HSD test (*p* < 0.05). All analyses were conducted in Stata 18.0 (Standard Edition). 

## Data Availability

The datasets used and/or analysed during the current study are available from the corresponding author upon reasonable request.
